# The Path to Functional Recovery After Thalamic Hemorrhage in a Young Adult: A Case Report

**DOI:** 10.7759/cureus.113449

**Published:** 2026-07-27

**Authors:** Tiffany T Nguyen, Hannah Oteng-Quarshie, Tha Cha

**Affiliations:** 1 Physical Medicine and Rehabilitation, California Health Sciences University, Clovis, USA; 2 Biomedical Education, California Health Sciences University, Clovis, USA; 3 Physical Medicine and Rehabilitation, San Joaquin Valley Rehabilitation Hospital, Fresno, USA

**Keywords:** hemorrhage, inpatient rehabilitation, neurological deficit, neuroplasticity, recovery of function

## Abstract

Intracerebral hemorrhage in young individuals is uncommon and often associated with significant neurologic morbidity. We present the case of an 18-year-old female patient with a spontaneous right thalamic hemorrhage with intraventricular extension, hydrocephalus, and subfalcine herniation requiring emergent neurosurgical intervention. Following medical stabilization, she underwent acute inpatient rehabilitation and progressed from total assistance for mobility and activities of daily living to ambulating with contact guard assistance over a 22-day course, with concurrent improvement in cognitive function. This case highlights the role of neuroplasticity and early multidisciplinary rehabilitation in facilitating meaningful functional recovery following severe intracerebral hemorrhage.

## Introduction

Intracerebral hemorrhage is a life-threatening neurologic event that is more commonly observed in older populations with established vascular risk factors [1]. In younger individuals, spontaneous hemorrhagic stroke is rare and often presents a diagnostic challenge due to a broader differential that includes vascular malformations, coagulopathies, and other less common etiologies as well as significant risk factors such as hypertension [2,3]. Beyond acute management, these cases provide a unique opportunity to examine patterns of functional recovery, particularly the role of neuroplasticity and early multidisciplinary rehabilitation in optimizing outcomes.

This report presents the case of a young adult with a spontaneous intracerebral hemorrhage resulting in significant neurologic deficits who demonstrated substantial functional recovery during an acute inpatient rehabilitation course. This case highlights the interaction between neuroplasticity and structured rehabilitation, emphasizing a function-centered approach to recovery in Physical Medicine and Rehabilitation (PM&R).

## Case presentation

An 18-year-old female patient with no prior functional limitations presented with a spontaneous right thalamic intraparenchymal hemorrhage with intraventricular extension, resulting in mild hydrocephalus and subfalcine herniation. The patient underwent neurosurgical intervention on hospital day 1 with decompressive craniectomy and external ventricular drain (EVD) placement, followed by EVD removal three days later.

Initial diagnostic workup raised concern for an underlying vascular malformation; however, subsequent angiographic evaluation revealed no clear source of hemorrhage. A small 2 × 2.3 mm intracranial aneurysm was identified but was considered incidental. Venography demonstrated patency of the major cerebral venous structures, with dominant left transverse and sigmoid sinuses and a hypoplastic but patent right proximal internal jugular vein. No D-dimer was obtained. The patient denied illicit drug use and was not taking any medications, including oral contraceptive pills. Urine toxicology screening was not performed.

Her hospital course was marked by significant neurologic deficits, including left-sided hemiplegia, facial weakness, and cognitive impairment affecting attention, memory, and executive functioning. Additional complications included bilateral upper extremity deep vein thromboses, anemia, and labile blood pressure requiring medical management.

Following medical stabilization, the patient was transferred to an acute inpatient rehabilitation facility approximately two weeks after initial neurosurgical intervention. At the time of rehabilitation admission, she required total assistance for mobility and activities of daily living, with marked left-sided weakness, impaired motor control, and significant visuospatial deficits including left neglect. Neurologic examination demonstrated asymmetric weakness, with right upper extremity strength approximately 4/5 and left upper extremity strength ranging from 2/5 to 3/5, consistent with left-sided hemiplegia. Lower extremity weakness was also present, more pronounced on the left.

The patient participated in an intensive rehabilitation program consisting of approximately three hours of therapy per day, including physical therapy, occupational therapy, and speech-language pathology. Physical therapy focused on mobility, transfer training, gait training, and balance activities. Occupational therapy emphasized activities of daily living and functional independence, while speech-language pathology addressed cognitive deficits including attention, executive functioning, and compensatory strategies. Rehabilitation goals were adjusted based on the patient's functional progress and evolving clinical needs.

Over the course of her 22-day inpatient rehabilitation stay (Table 1), the patient demonstrated substantial functional improvement. Early in her rehabilitation course, she required total assistance and was unable to safely ambulate. However, by the time of discharge, she progressed to ambulating approximately 250 feet with contact guard assistance using a front-wheeled walker and was able to perform activities of daily living with supervision to contact guard assistance.

**Table 1:** Functional progression during inpatient rehabilitation Functional status improved from total assistance to contact guard assistance over the course of inpatient rehabilitation, with concurrent improvement in cognitive function.

Timepoint	Mobility	Activities of daily living	Cognition
Admission	Total assist; non-ambulatory	Total assist	Severe deficits; left neglect
Mid-rehabilitation	Minimal assist transfers	Improving	Moderate deficits
Discharge	Approximately 250 ft ambulation with contact guard assistance (front-wheeled walker)	Supervision-contact guard assistance	Mild-moderate deficits

Detailed functional outcomes further illustrate the patient's progression in both mobility and self-care domains (Tables 2-3). These outcomes were assessed using Section GG functional scores, a standardized measure used in post-acute care settings to evaluate mobility and self-care performance, with lower scores indicating greater levels of assistance required [4]. At admission, several mobility tasks, including ambulation and transfers, were not attempted due to safety concerns. By discharge, the patient improved to supervision or contact guard assistance for sit-to-stand transitions, transfers, and ambulation at both 10- and 50-foot distances (Table 2).

**Table 2:** Mobility outcomes during inpatient rehabilitation Section GG functional scores: 01: dependent; 02: substantial/maximal assistance; 03: partial/moderate assistance; 04: supervision or touching assistance; 05: setup or clean-up assistance; 06: independent; 07: patient/resident refused; 09: not applicable; 10: not attempted due to environmental limitations; 88: not attempted due to a medical condition or safety concerns [4].

Activity	Admission	Discharge
Bed mobility	88	88
Sit to stand	02	04
Transfers	02	04
Walk 10 ft	88	04
Walk 50 ft	88	04

**Table 3:** Self-care outcomes during inpatient rehabilitation Section GG functional scores: 01: dependent; 02: substantial/maximal assistance; 03: partial/moderate assistance; 04: supervision or touching assistance; 05: setup or clean-up assistance; 06: independent; 07: patient/resident refused; 09: not applicable; 10: not attempted due to environmental limitations; 88: not attempted due to a medical condition or safety concerns [4].

Activity	Admission	Discharge
Eating	05	05
Oral hygiene	01	04
Upper body dressing	01	05
Lower body dressing	01	04
Toileting	01	04

Similarly, self-care performance improved substantially over the rehabilitation course. At admission, the patient required dependent assistance for most activities of daily living, including oral hygiene, dressing, and toileting. By discharge, she progressed to supervision or setup-level assistance, reflecting increased independence in daily functional tasks (Table 3).

Cognitive function also improved over time, with persistent but reduced deficits in executive functioning and attention. The Brief Interview for Mental Status (BIMS) was utilized to assess cognitive status and establish a baseline measure of cognitive function, serving as a brief screening tool commonly used in rehabilitation and post-acute care settings [5,6]. On formal assessment, the patient had a BIMS score of 15 on initial evaluation and 13 at discharge, consistent with mild to moderate cognitive impairment in the context of ongoing recovery. Although the BIMS score decreased slightly from 15 to 13, this may reflect variability in formal assessment and does not fully capture the patient's observed functional cognitive improvement, including increased use of compensatory strategies such as attention cueing and memory aids, which contributed to improved task performance and functional independence. She was discharged home with 24-hour caregiver support and plans for continued outpatient rehabilitation.

## Discussion

This case highlights key considerations in the management and recovery of hemorrhagic stroke in a young patient, including differences in stroke subtype, prognostic implications, and the role of neuroplasticity in functional recovery.

This case represents a hemorrhagic stroke, specifically an intracerebral hemorrhage involving the thalamus. Compared to ischemic stroke, hemorrhagic stroke is associated with greater early morbidity due to mass effect, increased intracranial pressure, and secondary injury to surrounding brain tissue [7]. In this patient, intraventricular extension, hydrocephalus, and subfalcine herniation reflect a high initial injury burden, which is typically associated with poorer early functional outcomes [7].

Despite the severity of her presentation, this patient demonstrated substantial functional recovery over a relatively short period of rehabilitation. Functional recovery following stroke is influenced by lesion location, initial severity, and patient-specific factors such as age. Younger patients, in particular, may experience more favorable outcomes due to increased neuroplastic capacity [8], with this population often presenting with decreased burden of disease [9]. Although thalamic involvement can result in significant motor and cognitive deficits [10], recovery in younger individuals may be enhanced by preserved neural network adaptability and the ability to recruit alternative pathways [10].

The observed improvements in both motor function and cognitive performance in this patient underscore the role of neuroplasticity in post-stroke recovery. Neuroplasticity refers to the brain's ability to reorganize and form new neural connections in response to injury [11]. This process is driven by repetitive, task-specific training and is most active during the early recovery period [12]. The patient's transition from complete dependence to supervised functional mobility suggests robust early neuroplastic adaptation facilitated by intensive rehabilitation. These findings further support the observed transition from dependence to supervision-level assistance across functional domains (Tables 2-3).

Importantly, this recovery extended beyond motor domains. The patient demonstrated measurable improvement in executive functioning, attention, and visuospatial awareness, though residual deficits persisted. The presence of left-sided neglect and executive dysfunction highlights the importance of addressing higher-level cognitive impairments, which can significantly impact safety and independence even when motor recovery is achieved. Targeted cognitive rehabilitation strategies, including the use of metacognitive techniques and external supports, likely contributed to her functional gains.

This case reinforces the critical role of early, intensive, and multidisciplinary rehabilitation in driving functional recovery, with greater outcomes noted in strokes of higher severity [13]. The integration of physical, occupational, and speech therapy allowed for the simultaneous targeting of motor, cognitive, and functional deficits [1], aligning with core PM&R principles focused on restoring independence and quality of life. Early engagement in rehabilitation has been shown to enhance neuroplastic processes and improve long-term outcomes, particularly in younger patients with greater recovery potential [10-12] as well as the importance of avoiding pessimistic prognostication during the first year of recovery [14]. This case is notable for the substantial functional recovery observed in a young adult with thalamic hemorrhage despite significant initial neurologic deficits, highlighting the potential influence of neuroplasticity and comprehensive inpatient rehabilitation on recovery outcomes.

A limitation of this report is that the original neuroimaging studies were unavailable for direct review during manuscript preparation; therefore, the imaging findings presented are based on the official radiology reports. An additional limitation is that urine toxicology screening was not performed. Although the patient denied illicit drug use, stimulant-associated intracerebral hemorrhage remains an important diagnostic consideration in young patients presenting with spontaneous hemorrhage, particularly in the absence of an identifiable etiology.

Although the etiology of this patient's hemorrhage remained unclear despite angiographic evaluation, her clinical course demonstrates that meaningful functional recovery is achievable even in the setting of severe initial injury. This reinforces the importance of focusing not only on diagnosis and acute management but also on rehabilitation and functional prognosis, particularly in younger populations.

## Conclusions

This case illustrates that substantial functional recovery is achievable following a high-burden intracerebral hemorrhage in a young patient, even in the absence of an identifiable etiology. The patient's progression from total dependence to supervised functional mobility over a short inpatient rehabilitation course reflects clinically meaningful improvement in both motor and cognitive domains. These outcomes underscore the critical role of early, intensive, multidisciplinary rehabilitation in facilitating recovery through neuroplastic adaptation. In the PM&R setting, attention to both physical and cognitive deficits, along with the integration of compensatory strategies, is essential in optimizing independence and long-term functional outcomes. This case reinforces the importance of a function-centered approach in stroke management, particularly in younger patients with greater neuroplastic potential.
